# Plasticity in the Brainstem: Prenatal and Postnatal Experience Can Alter Laterodorsal Tegmental (LDT) Structure and Function

**DOI:** 10.3389/fnsyn.2020.00003

**Published:** 2020-02-07

**Authors:** Kristi A. Kohlmeier, Filip S. Polli

**Affiliations:** Department of Drug Design and Pharmacology, University of Copenhagen, Copenhagen, Denmark

**Keywords:** drug dependency, REM sleep, mesopontine cholinergic, nitric oxide synthase, plasticity, laterodorsal tegmental nucleus

## Abstract

The brainstem has traditionally been considered an area of the brain with autonomous control of mostly homeostatic functions such as heart rate, respiration, and the sleep and wakefulness state, which would preclude the necessity to exhibit the high degree of synaptic or cellular mechanisms of plasticity typical of regions of the brain responsible for flexible, executive control, such as the medial prefrontal cortex or the hippocampus. The perception that the brainstem does not share the same degree of flexibility to alter synaptic strength and/or wiring within local circuits makes intuitive sense, as it is not easy to understand how “soft wiring” would be an advantage when considering the importance of faithful and consistent performance of the homeostatic, autonomic functions that are controlled by the brainstem. However, many of the molecular and cellular requirements which underlie strengthening of synapses seen in brain regions involved in higher-level processing are present in brainstem nuclei, and recent research suggest that the view of the brainstem as “hard wired,” with rigid and static connectivity and with unchanging synaptic strength, is outdated. In fact, information from studies within the last decades, including work conducted in our group, leads us to propose that the brainstem can dynamically alter synaptic proteins, and change synaptic connections in response to prenatal or postnatal stimuli, and this would likely alter functionality and output. This article reviews recent research that has provided information resulting in our revision of the view of the brainstem as static and non-changing by using as example recent information gleaned from a brainstem pontine nucleus, the laterodorsal tegmentum (LDT). The LDT has demonstrated mechanisms underlying synaptic plasticity, and plasticity has been exhibited in the postnatal LDT following exposure to drugs of abuse. Further, exposure of the brain during gestation to drugs of abuse results in alterations in development of signaling pathways in the LDT. As the LDT provides a high degree of innervation of mesoaccumbal and mesocortical circuits involved in salience, as well as thalamocortical circuits involved in control of arousal and orientation, changes in synaptic strength would be expected to alter output, which would significantly impact behavioral state, motivated behavior and directed attention. Further, alterations in developmental trajectory within the LDT following prenatal exposure to drugs of abuse would be expected to impact on later life expression of motivation and arousal.

## The Laterodorsal Tegmental Nucleus Has a Prominent Role in Control of the Sleep and Wakefulness Cycle (Introduction)

The laterodorsal tegmentum (LDT) is located in the pontine central gray, just below the caudal portion of the aqueduct, which connects the third and fourth ventricles. The LDT is a heterogeneous nucleus composed of cholinergic, GABAergic and glutamatergic neurons (Vincent et al., 1983; Clements and Grant, 1990; Wang and Morales, 2009). The efferent projections from the LDT stream both rostrally and caudally. Rostral projections include terminations within the thalamus, hippocampus and basal forebrain, whereas, caudal pathways terminate within several nuclei within the pons and medulla (Woolf and Butcher, 1986). Lesion studies in the 1960s revealed the critical role of the LDT in control of the states of sleep and wakefulness (Jouvet, 1965). Follow up work in the 1980s and the 1990s revealed that it was cholinergic neurons of the LDT which were vital for this function, and optogenetic studies in the 2010’s have validated the important role of the cholinergic cells in this control (Baghdoyan et al., 1987; Grant and Highfield, 1991; Kayama et al., 1992; van Dort et al., 2015). The cholinergic neurons of the LDT comprise part of the reticular activating system, which is a collection of brainstem nuclei, including the locus coeruleus (LC) and dorsal raphe (DR), which collectively project to the thalamus where they participate in gating of cortical activity underlying states of wakefulness, arousal, and the stages of sleep (Baghdoyan et al., 1987; Steriade et al., 1990). In addition, the cholinergic neurons of the LDT have been shown to play a particular role in gating and controlling rapid eye movement sleep (REM sleep), which is a state when LC and DR neurons are silent (McGinty and Harper, 1976; Aston-Jones and Bloom, 1981; Mansari et al., 1989). Paradoxically, activity of a population of the cholinergic cells of the LDT is highest in REM and during wakefulness, with the lowest activity expressed during slow wave sleep (Boucetta and Jones, 2009; Boucetta et al., 2014). How activity within the same neuronal population can participate in such diametrically opposed behavioral states remains a great source of investigation, but the high activity might involve generation of cortical activity promotive of processing, encoding, and storage of waking life experiences dependent on synaptic strengthening, however, the synaptic homeostasis theory of the function of sleep posits that sleep is a time of synaptic scaling resulting in synaptic weakening (Poe, 2017; Tononi and Cirelli, 2019).

## The Known Role of the LDT Expands

**FIGURE 1:**
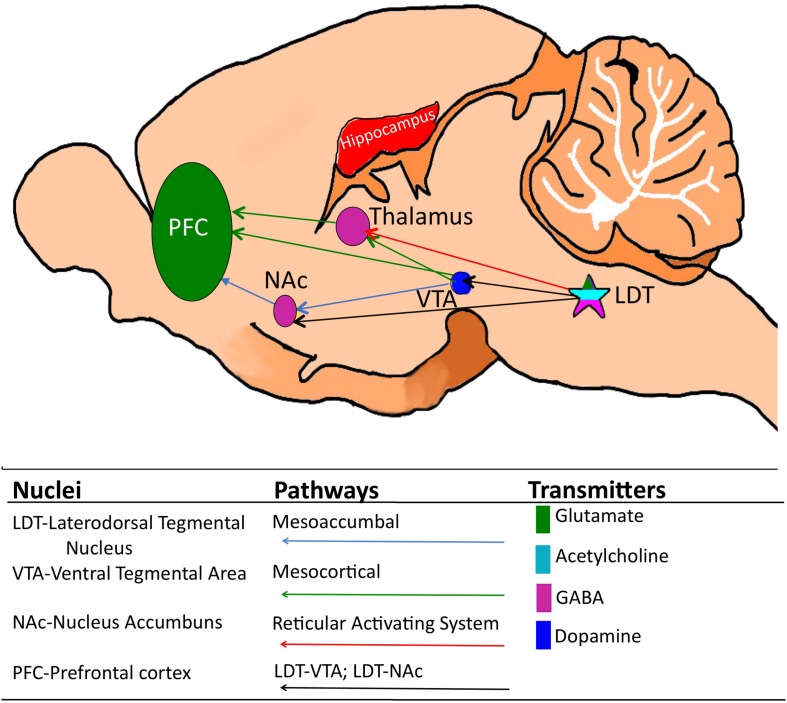
Cartoon of the mouse brain showing relevant corticothalamic, mesoaccumbal and mesocortical pathways and, while not an exhaustive list, the relevant key neuronal phenotypes. The laterodorsal tegmentum (LDT) which is comprised of glutamate, GABA and acetylcholine-containing neurons is located in the pons and sends projections to a multiplicity of brain sites, including the thalamus, the ventral tegmental area (VTA) and the nucleus accumbens (NAc). Via these projections, the LDT governs arousal associated with learning and memory and motivated behaviors, including drug seeking behavior, as well as cortical electrical signatures of arousal.

Although outside of the scope of the early work focusing on the role of the LDT in state control, anatomical tract tracing studies showed that the LDT does not just project upstream to the thalamus, where it controls cortical neuronal firing patterns typical of alert wakefulness and REM sleep, or downstream to pontine and medullary nuclei controlling the classic, motor-related physiological sign of REM sleep, motor atonia (Sofroniew et al., 1985; Cornwall et al., 1990; Steriade et al., 1990; Jones, 1991; Imon et al., 1996). Rather, the LDT projects to a multiplicity of sites, including cortical, sensory, and limbic structures (Satoh and Fibiger, 1986; Woolf and Butcher, 1986; Cornwall et al., 1990). Many of these neuronal sites were not easily incorporated into the classic functional circuits known to play a role in control of states of wakefulness or sleep. Therefore, their presence suggested a role of the LDT beyond state control, however, the functional significance of these other projections was not studied until the mid to late 1990s. One major projection terminates in the ventral tegmental area (VTA) and the nucleus accumbens (NAc), which are central players in the mesoaccumbal and mesocortical pathways of the limbic system (Figure 1). Together with the prefrontal cortex, the VTA and NAc comprise the brain’s reward circuitry and activity involving alterations in dopamine (DA) within the mesoaccumbal pathway in which they participate is generated by pleasurable and aversive stimuli (Di Chiara and Imperato, 1988; Damsma et al., 1992; Hansen et al., 1993; Kiyatkin and Gratton, 1994).

As the VTA was known to be important in goal-directed behavior based on seminal studies from the 1970’s, identification of the projections to the mesoaccumbal pathway had raised speculation that the LDT could be involved in limbic processing of behaviorally-relevant stimuli (Cornwall et al., 1990; Oakman et al., 1995). Investigations of these projections using functional techniques were conducted. Chronoamperometry revealed that electrical stimulation of the LDT resulted in phasic rises of DA in the NAc (Forster and Blaha, 2000). An earlier study using pharmacological stimulation had demonstrated a role for LDT cholinergic mechanisms in NAc DA rises (Blaha et al., 1996), which was also seen in the electrical stimulation studies, and further, contribution of a glutamatergic component sourcing from the LDT was also noted (Forster and Blaha, 2000). Investigations of the structural organization of the LDT showed that this nucleus provides glutamatergic and GABAergic synaptic inputs to neurons of the VTA and NAc (Omelchenko and Sesack, 2005; Wang and Morales, 2009). As many regions provide GABA and glutamatergic inputs to the VTA and NAc, this was not as profound as data indicating that the LDT, along with the neighboring pedunculopontine tegmentum (PPT), provides the primary cholinergic input to the mesoaccumbal region, suggesting a potential for domination of cholinergic modulation within these midbrain groups by the two acetylcholine (ACh)-containing brainstem nuclei (Cornwall et al., 1990; Oakman et al., 1995; Omelchenko and Sesack, 2005, 2006; Wang and Morales, 2009). Further, later work showed that cholinergic projections from the LDT are preferentially directed to the lateral region of the VTA, that projects to the lateral shell of the NAc (Lammel et al., 2012), which is a subregion importantly involved in motivating behaviors (Tsai et al., 2009; Witten et al., 2011; Pascoli et al., 2015). When taken together, the anatomical and physiological studies showed that the LDT-VTA-NAc pathway noted in the 1980’s was functional. Interestingly, what motivational stimuli share in common, whether they are pleasurable or aversive is their ability to arouse. Once the critical role of the LDT in the reticular activating pathway, and in control of the sleep and wakefulness state was recognized, that might have presaged formulation of the hypothesis of functional influence of the LDT on motivated states earlier than anatomical studies identified the presence of the LDT-VTA-NAc pathway.

### The VTA and the NAc Reward Pathway

After decades of study, the VTA-NAc pathway is now known to play a fundamental role in assignment of reward to stimuli, including reward assigned to drugs of abuse, and further, the idea that DA plays a critical role in this function is widely accepted. In the mid 1970s, findings that DA receptor blockers reduced lever press for intravenous amphetamine self-administration in rats led to the suggestion that DA plays a central role in perception and extent of reward (Yokel and Wise, 1975). Studies conducted at the end of the 1980s were among the first to present a common biochemical basis of the rewarding effects of different classes of drugs of abuse. These investigations employed microdialysis methods to measure extrasynaptic levels of DA in different brain areas, including the NAc. Following peripheral administration of all drugs of abuse tested (amphetamine, cocaine, morphine, ethanol, and nicotine), DA levels in the NAc of freely moving rats were found to exceed basal levels by over 100% and remain elevated for hours, which exhibited a dependency upon drug concentration (Di Chiara and Imperato, 1988).

Further work showed that electrical stimulation of VTA neurons enhanced cueing effects of amphetamine (Druhan et al., 1990), while activation of type 1 DA receptors (D1) in the NAc increased cocaine self-administration and produced conditioned place preference (CPP), which is a behavioral indicator of the rewarding effects of drugs of abuse (White et al., 1991; Robledo et al., 1992). These and other studies lead researchers to propose activation of DA neurons of the VTA, with subsequent release of DA in targets in the NAc, as a central mechanism signaling rewarding effects of drugs of abuse (Di Chiara and Imperato, 1988). Reward reinforcement of behaviors required for continuation of the species, such as sex (Damsma et al., 1992), social interactions (Hansen et al., 1993), and consumption of food (Kiyatkin and Gratton, 1994), were also shown to be mediated by DA VTA-NAc signaling.

Several sources of modulation within the VTA have been shown to lead to drug-induced DA rises. Drugs of abuse exhibit a variety of pharmacological mechanisms, and rises in DA can occur via actions in the synaptic cleft, direct excitation of DA cells, and/or via activation of non-DA VTA cells (Nestler, 2005; Lüscher and Ungless, 2006; Pierce and Kumaresan, 2006). Although there is some debate as to exact proportions, GABA cells comprise the largest population of non-DA VTA cells (Johnson and North, 1992; Margolis et al., 2006), with some contribution from glutamate cells (Nair-Roberts et al., 2008; Yamaguchi et al., 2015). The non-DA cells have been shown to respond to drugs of abuse differentially dependent on drug class with a complex impact on intra VTA circuit dynamics likely affecting encoding of stimuli valence (Hnasko et al., 2012; Yan et al., 2019; Grieder et al., 2019). Drugs not abused by humans, such as imipramine and atropine, fail to induce DA rises in the NAc, while drugs with aversive properties, including κ-opioid agonists, can reduce DA levels within this region, however, interestingly, enhanced activity of DA VTA cells has also been implicated in aversion to nicotine (Di Chiara and Imperato, 1988; Grieder et al., 2019).

Recent development of optogenetic tools allowed further characterization of DA VTA neurons which has shown that they display reward-related firing patterns, and that their activity is sufficient to trigger addiction-related behaviors, the later shown even in the absence of any drug exposure (Tsai et al., 2009; Witten et al., 2011; Pascoli et al., 2015). Taken together, these studies support the conclusion that DA signaling within the NAc, sourcing from VTA neurons plays a key role in the processing of experience-driven reward. Firing of these cells encodes motivational salience which turns an organism toward, or away, from repetition of behaviors leading to acquisition of a stimulus. While this is a necessary process to encourage repetition of healthy behaviors in order to ensure our existence, when the activity is promoted by drug activity, this can be counterproductive. However, while the VTA is critical in signaling salience to natural and drug-related stimuli, it does not work in isolation to encode reward valence in motivational circuits, since DA VTA neuronal firing is strongly influenced by afferent input, which might itself be enhanced by natural or drug-related stimuli.

## LDT-VTA Inputs

### *In vivo* and Pharmacologic Studies of Afferent Input From the LDT to the VTA

The anatomical connectivity from the LDT to the VTA suggested that the LDT likely participates in motivated and salience-related behaviors. Indeed, a functional role of the LDT-VTA pathway in addiction-related behaviors has been corroborated by several *in vivo* and *in vitro* approaches (Satoh and Fibiger, 1986; Cornwall et al., 1990; Oakman et al., 1995). Amphetamine-induced locomotion and behavioral sensitization were shown to be reduced in LDT lesioned rats, suggesting regulation by the LDT of VTA output of DA (Laviolette et al., 2000). Consistent with this, LDT stimulation in anesthetized rats increased DA release into the NAc, which was potentiated by i.p. injections of cocaine (Lester et al., 2010). These effects were attenuated by intra-VTA application of scopolamine, a blocker of mAChRs, suggesting that cholinergic projections in the LDT-VTA pathway are involved in enhancement of accumbal DA release (Lester et al., 2010). However, the role of the LDT in enhancement of DA levels in the striatum might vary depending on the drug class or exposure protocols. While LDT-lesioned rats showed attenuation of DA levels in response to morphine, enhanced DA levels was shown in the same striatal region following administration of amphetamine which was half of the concentration utilized earlier in examination of amphetamine actions in presence of LDT lesion (Forster et al., 2002). Blockade of glutamatergic AMPA receptors in a region of the brainstem encompassing the LDT, which should inhibit LDT excitability, was shown *in vivo* to attenuate reinstatement of drug seeking induced by cocaine injection, an effect also observed after intra-VTA blockage of AMPA, nAChR, and mAChR receptors, suggesting that ACh output from the LDT was involved, with perhaps a contribution from glutamatergic LDT output, and further, that excitatory glutamatergic activity within the LDT was important for drug induced behaviors (Schmidt et al., 2009). A role for glutamatergic activity in the LDT in drug-addiction-associated behaviors was also indicated by intra-LDT pharmacologic blockade of AMPA and NMDA receptors *in vivo* which attenuated cocaine CPP (Shinohara et al., 2014; Kaneda, 2019). This attenuation reached its maximum effect following intra-LDT application of carbachol, which hyperpolarizes LDT neurons when acting at mAChRs, leading to a profound inhibition (Shinohara et al., 2014; Kaneda, 2019). Finally, a study of the role of the functional significance of the LDT-VTA pathway in addiction-related behaviors and signaling provided probably the first indication that drug-related plasticity can occur in the LDT, albeit this was not the focus of the study. AMPA injection in the LDT lead to sustained enhancements in glutamate recorded in the VTA as well as rises in DA in the NAc in amphetamine-treated rats (Nelson et al., 2007). Interestingly, significant rises in glutamate released in VTA were seen following intra LDT injection of AMPA in non-amphetamine treated controls as well (Nelson et al., 2007). While this did indicate the presence of plasticity in the LDT-VTA pathway leading to heightened LDT output to mesoaccumbal structures, an effect which was termed “LTP-like,” direct evidence of the underlying cellular mechanism was not provided as this was beyond the scope of the work (Nelson et al., 2007).

Although these *in vivo* studies certainly contributed to the understanding that the LDT is involved in the circuits critical to dependency, they were technically limited by their inability to demonstrate LDT direct actions in the VTA since effects following nucleus-specific manipulations, including intra-nucleus injections, could have been indirect or included targets beyond those intended. However, evidence of direct involvement of LDT-VTA pathways in motivated behaviors and cellular activity was provided by studies employing optogenetic approaches. Specific opto-stimulation of LDT inputs into the VTA was shown to elicit CPP in mice (Lammel et al., 2012), and photoexcitation of LDT specific cholinergic projections within the VTA was associated with positive reinforcement as CPP was induced (Xiao et al., 2016). Interestingly, in a combined behavioral and optogenetic study which was notable because LDT-VTA cholinergic and glutamatergic terminals were selectively activated under the same experimental conditions allowing a direct comparison of effect, cholinergic input was found to be critical in a CPP assay for reinforcement of remaining in the conditioned chamber, however, glutamate release was determined to be involved in initial entries into the conditioned chamber, suggesting complementary, but separate functions of the two different LDT populations (Steidl et al., 2017).

While the preponderance of studies have shown a role of the LDT in reinforcement of relevant stimuli indicative of a positive valence, activity in the LDT-VTA circuits has also been shown to be involved in coding negative valence underlying aversion. The precise local circuits and pathways involved in coding aversion, however, are not fully elucidated. High doses of nicotine can be aversive (Goldberg et al., 1981, 1983; Goldberg and Spealman, 1982, 1983) which has been shown to involve stimulation of the medial habenula-interpeduncular (IPN) pathway (Fowler and Kenny, 2014). Interestingly, while an earlier study suggested distinct populations of VTA neurons involved in encoding reinforcement and avoidance, with the LDT to VTA projection involved in positive encoding and a projection from the lateral habenula to the VTA regulating aversion (Lammel et al., 2012), a later optogenetic study revealed a role of the LDT in avoidance encoding to aversive doses of nicotine that involved activation of IPN-sourced GABAergic inputs directed to phenotypically-unidentified neurons of the LDT, which projected to the VTA (Wolfman et al., 2018). Further, while not strictly an aversion-inducing stimuli, social defeat stress was associated with hyperactivity of cholinergic and glutamatergic LDT neurons which projected to the VTA, however, further studies indicated that of the two cell types, only cholinergic neurons were involved in maladaptive responses to stress (Fernandez et al., 2018). Although caution in making conclusions was noted, stimulation of LDT cholinergic afferents to DA-VTA neurons was found to enhance the increase in firing noted upon an aversive, paw pinch stimulation (Dautan et al., 2016). While it appears a role is played by GABAergic neurons of the LDT in encoding aversion to olfactory innate fear, and optogenetic activation of this cell type induced fear and anxiety-like behaviors, these actions were not believed to involve subsequent activation of the VTA, and accordingly, different pathways were suggested (Yang et al., 2016). At this time, dissection of the role played by the different LDT neuronal phenotypes in negative reinforcement and stress, as well as elucidation of the mechanisms which can explain how cholinergic, glutmatergic, and GABAergic output from the LDT to likely different populations of DA, and non-DA, cells of the VTA can be involved in encoding both positive and negative valence remain an open area of investigation.

While focus of LDT involvement in studies of motivated behaviors and the role of DA release in these behaviors has been most commonly on cholinergic and glutamatergic input from the LDT to the VTA, direct projections from the LDT to the NAc have been characterized (Dautan et al., 2014). Recently, *in vivo* optogenetic activation of cholinergic inputs sourcing from the LDT and synapsing within the NAc were found to shift preference, enhance motivation, and drive positive reinforcement in drug-related behavioral tests (Coimbra et al., 2019). This finding suggests that LDT involvement in induction of salience can occur independent of LDT modulation of VTA dopaminergic function, which raises the complexity by which the LDT can control output of the mesoaccumbal pathway.

### Cellular Studies of the LDT-VTA Pathway

The effect on DA VTA cell activity and output of stimulation of the LDT-VTA pathway has been examined *in vivo* and *in vitro*, and absence or presence of activity in this pathway was found to explain some conflicting data in the literature using these two approaches. The LDT has been shown to regulate activity of DA VTA cells and the pattern of this regulation likely plays a major role in behavioral outcomes. Activation of the LDT nucleus in anesthetized rats revealed an interesting modulation of DA release into the NAc, in which three components were reliably and reproducibly evoked: (1) brief increase in DA release; (2) followed by a decreased DA release below baseline levels; and (3) a sustained DA release for several minutes, which was at levels approximately 80% higher than baseline. The blockage of nicotinic receptors (nAChRs) or ionotropic glutamate receptors in the VTA attenuated the first component, while suppression of the second component was achieved by intra-LDT blockage of muscarinic receptors (mAChRs) suggesting an autoregulatory role. Intra-VTA infusions of mAChR blockers were shown to attenuate the third, sustained component (Forster and Blaha, 2000). When taken together, these data suggested that DA levels in the NAc are selectively mediated by LDT-elicited activation of glutamatergic, nAChR, and mAChR in the VTA, as well as mAChRs on LDT neurons (Forster and Blaha, 2000). These findings are aligned with previous data showing that both glutamatergic and cholinergic LDT neurons establish multiple, asymmetric, presumably excitatory, contacts with DA-containing VTA cells (Cornwall et al., 1990; Oakman et al., 1995). Fast, direct excitation of DA VTA cells likely occurs at demonstrated postsynaptically-located ionotropic AMPA and nACh receptors (Di Chiara and Imperato, 1988; Forster and Blaha, 2000; Lammel et al., 2012). However, ACh-stimulated excitation is also likely mediated by metabotropic mAChRs. Following earlier studies showing that the DA VTA neuronal population is one of the few in the brain expressing M_5_-type mAChRs (Weiner et al., 1990), DA release was shown to be stimulated by LDT-mediated cholinergic excitation of M_5_-type mAChRs (Forster and Blaha, 2000; Miller and Blaha, 2005; Lester et al., 2010). The conclusion that activation of M_5_ mAChRs leads to sustained increases in DA release within the NAc is supported by a study showing that knockout mice for the M_5_ mAChR failed to demonstrate the long-lasting, third component of DA release in the NAc upon LDT-activation (Forster et al., 2002), and by a more recent investigation demonstrating that pharmacological activation of M_5_ receptors on VTA neurons potentiates DA release into the striatum (Shin et al., 2015). Another subtype of mAChR also plays a role in DA VTA functioning, as M_4_ receptors located on LDT terminals in the VTA were shown to play an autoregulatory function, and limit release of ACh in the VTA (Zhang et al., 2002).

*In vivo*, behaviorally-relevant release of DA into the NAc occurs upon a burst-firing pattern, which has been shown to rely on an intact LDT, and it was this recognition that reconciled conflicts in the literature that burst firing of VTA cells could be elicited *in vivo*, but not *in vitro* (Lodge and Grace, 2006). The role of the LDT in DA VTA burst firing was reinforced, and refined by optogenetic studies showing that activation of cholinergic LDT projections can alter VTA DA firing such that ongoing firing activity can be shifted, resulting in greater intensity of bursting, presumably resulting in heightened DA output to the NAc (Dautan et al., 2016). In addition, this approach revealed that LDT cholinergic and glutamatergic neurons can modulate firing activity of both DA and non-DA VTA neurons (Dautan et al., 2016). Interestingly, behavioral actions of endogenous peptides which induce arousal, such as those important in appetite control and food seeking behavior, of which ghrelin and orexin are excellent examples, rely on cholinergic tone in the VTA for their activation of DA VTA cells, and, this tone was demonstrated to act at VTA nACh receptors (Borgland et al., 2006; Jerlhag et al., 2012). Both orexin and ghrelin have been shown to induce excitation of LDT neurons, which would be expected to trigger output from the LDT to target regions (Kohlmeier et al., 2004; Takano et al., 2009; Hauberg and Kohlmeier, 2015). In support of this conclusion, ghrelin has been shown to result in concomitant rises in ACh in the VTA and DA in the NAc (Jerlhag et al., 2012). Finally, although the VTA and DA are not incorporated into theories of sleep control circuits, optogenetic evidence suggests revision of this lack of inclusion as an active VTA counters initiation of sleep, and an inhibitory effect on REM sleep was noted (Eban-Rothschild et al., 2016), supporting the interpretation that excitatory afferent input occurs during wakefulness. From all of the studies of the LDT-VTA pathway, a picture has emerged that cholinergic and glutamatergic output from the LDT to the VTA is essential in controlling behaviorally-relevant VTA firing. Actions appear to be direct on DA VTA cells, but as non-DA VTA cells also contain ACh and glutamate receptors, and receive input from the LDT, modulation of their activity following LDT stimulation is also likely involved, but the precise contribution of LDT-stimulated activity of non-DA VTA cells in control of motivated behavior needs to be further elucidated (Mansvelder and McGehee, 2000; Mansvelder et al., 2002; Lammel et al., 2012; Dautan et al., 2014, 2016; Yan et al., 2018, 2019). When taken together, the role of the LDT in activation of mesoaccumbal, with participation also of activity within mesocortical, pathways seems to include stimulation of arousal with the likely outcome of directing orientation and focus of the individual toward the relevant, triggering environmental or internal stimulus. Interestingly, this suggests that the LDT cholinergic inputs to the VTA could exhibit projections and/or firing patterns distinct from those cholinergic LDT cells which exhibit high firing levels during REM sleep.

## Cellular and Synaptic Mechanisms Underlying Plasticity

Mechanisms underlying the molecular and cellular basis of learning and memory remain one of the most popular investigative topics in neuroscience because these processes are central to shaping what an individual is, and how they are perceived by others. Understanding the underlying mechanisms of these cognitive-related processes is expected to reveal how the brain actually changes in response to day-to-day experiences. In addition, elucidation of underlying mechanisms of these operations has relevance for understanding and treating disease states which present with alterations in cognition manifested as decrements in learning and memory, such as Alzheimer’s disease. Synaptic plasticity is the ability of synapses to change their efficiency and is believed to underlie learning and memory. Several different forms of synaptic plasticity are likely at the heart of re-wiring of the brain in response to experiences. Although changes in the intrinsic properties of cell membranes that govern neuronal excitability have been associated with an activity-dependent, glutamate-independent form of plasticity, coined as intrinsic plasticity (Zhang and Linden, 2003; Lisman et al., 2018; Debanne et al., 2019), functional alterations in glutamatergic receptors seem to play a more central, mechanistic role in synaptic plasticity associated with information storage in cognition-related neural centers (Diering and Huganir, 2018).

A series of electrophysiological experiments conducted in the hippocampus suggested that increases in glutamatergic synaptic strength through both pre- and postsynaptic mechanisms occur, which has been coined Long Term Potentiation (LTP). Blockade of LTP *in vivo* has been shown to result in cognitive dysfunctions (Abel et al., 1997; Pastalkova et al., 2006; Sacktor, 2008). To date, LTP represents the best mechanistic explanation of the synaptic basis of memory (Bliss and Collingridge, 1993), and corroborates earlier predictions that activity-dependent, synaptic strengthening reflects learning and memory consolidation in the brain (Hebb, 1949). Postsynaptic NMDARs were found to drive most forms of LTP known (Bliss and Collingridge, 2013), and genetic manipulations that impair, or restrict, NMDA receptor functioning or expression induce deficits in learning and memory (Nakazawa et al., 2004). AMPAR activation represents the main mechanism of neuronal depolarization, which allows the Mg^+2^ unblocking of, and calcium signaling through, NMDARs during experience-driven activities. The activation of NMDAR-mediated events, in turn, recruits AMPARs to postsynaptic sites, in which strengthening of synaptic connections is reflected in higher amplitudes of AMPAR-mediated postsynaptic potentials and currents upon subsequent activation within that synapse (Malinow and Malenka, 2002; Kandel et al., 2013). Drugs of abuse have been well established to result in LTP at excitatory and inhibitory synapses in the VTA, which can be transient, and context specific. However, with chronic exposure, this “drug-evoked synaptic plasticity” likely contributes significantly to expression of drug-dependent behavior (for review, see Ungless et al., 2001).

Another mechanism of glutamate-related plasticity was recognized when it was discovered that some postsynaptic sites are composed of only one type of conditionally functional ionotropic glutamate receptor, the NMDAR. Due to the Mg^+2^ blockage, these synapses are not functional at resting membrane potential, as glutamate release from presynaptic sites is unable to induce appreciable levels of currents in the postsynaptic neuron upon binding to the NMDAR. These NMDAR-containing synapses which lack functional AMPAR, were termed “silent synapses” (Isaac, 2003). Silent synapses were found to be abundant during developmental stages (Durand et al., 1996; Isaac et al., 1997) however, their numbers decrease across ontogeny with a very low presence in adulthood, suggesting they play a role in brain circuit maturation. Upon continuous glutamate binding to postsynaptic NMDARs, these synapses were shown to undergo un-silencing processes through recruitment of functional AMPARs (Lee et al., 2013; Dong and Nestler, 2014; Ma et al., 2014). It has been suggested that un-silencing plays a role in establishing new synapses, rewiring brain circuits, and in contributing to LTP establishment (Malenka and Nicoll, 1997; Brecht and Feldman, 2005; Kerchner and Nicoll, 2008; Hanse et al., 2013; Dong and Nestler, 2014).

Interestingly, silent synapses can be generated *de novo* in the brain following certain experiences, such as upon exposure to drugs of abuse, where it is believed they play a role in remodeling reward-processing brain regions (Kerchner and Nicoll, 2008; Hanse et al., 2013; Whitaker et al., 2016; Beroun et al., 2018; McDevitt and Graziane, 2018). However, their appearance or activation upon drug exposure can be dependent on many factors. Acute nicotine application in hippocampal brain slices of newborn rats can activate silent synapses in CA1 neurons, whereas, chronic nicotine exposure in mice was associated with increased number of silent synapses in medium spiny neurons of the striatum (Maggi et al., 2003; Xia et al., 2017), suggesting that the outcomes of nicotine-induced alterations, and perhaps also of other drugs, on silent synapses likely depends on brain areas, drug concentration, and developmental phase at which drug exposure occurs. When taken together, silent synapses represent a powerful mechanism of metaplasticity in the brain, that can be either generated or unsilenced in a region-specific, age-dependent fashion and which appear to be dynamically controlled by drugs of abuse, which likely importantly contribute to drug-induced remodeling of reward-related circuits (Abraham and Bear, 1996; Brown et al., 2011; Lee et al., 2013; Huang et al., 2015; Dong, 2016).

## Evidence of Plasticity and Metaplasticity in the LDT

Given the role of LTP and silent synapses in learning and memory, induction and presence of these processes have been examined predominantly in forebrain regions with little focus on the brainstem, although AMPAR-present, silent synapses were shown postsynaptically in the nucleus tractus solitarii (Balland et al., 2008). Although plasticity in the LDT had been postulated based on behavior, microdialysis, and lesion studies (Nelson et al., 2007), and a mechanism of presynaptic plasticity was shown by recording identified cholinergic LDT neurons following chronic administration of cocaine which resulted in increases in glutamate release from presynaptic terminals that reversed after 5 days post treatment (Kurosawa et al., 2013), the ability to induce glutamate receptor-dependent postsynaptic LTP in the LDT, or the presence of NMDAR-containing silent synapses in this nucleus, had not been previously shown at the cellular level. However, recent studies from our lab have demonstrated that silent synapses are present in the LDT at juvenile stages (Polli and Kohlmeier, 2019), and further, that LTP can be induced in neurons of this nucleus.

**FIGURE 2:**
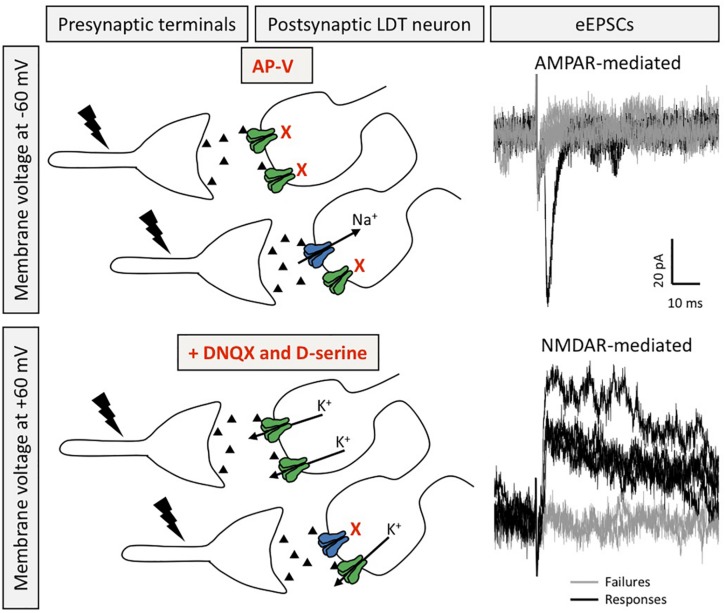
Silent synapses are present in LDT neurons. Silent synapses were identified in *ex vivo* brain slices from juvenile mice using the minimum stimulation protocol, in which several EPSCs are evoked (0.1 Hz) at resting membrane potential in the presence of the NMDAR blocker AP-V and with QX-314 in the patch pipette to block voltage gated sodium channels in order to isolate AMPAR-mediated responses in absence of elicitation of action potentials **(top)**. After washout of AP-V, in the presence of DNQX and D-serine, the voltage command is set to a depolarized membrane potential to evoke NMDAR-mediated EPSCs recorded as outward potassium currents **(bottom)**. Silent synapses are calculated by the comparison of the failure rate between NMDAR- and AMPAR-mediated eEPSCs. Recordings in black denote eEPSCs, whereas gray traces depict failures (for both conditions). Postsynaptic receptors shown in blue are AMPAR and those in green are NMDAR.

**FIGURE 3:**
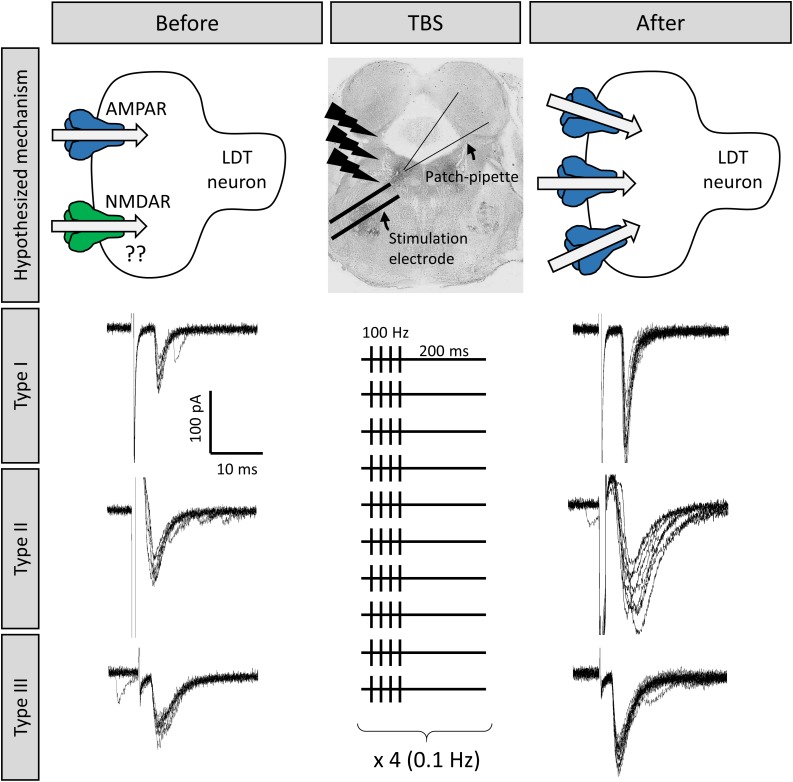
Long term potentiation (LTP) could be induced at LDT synapses, which was an effect seen in all three cell types. Shown are the responses of three different LDT neurons representative of the three electrophysiological cell types in the LDT to electrical stimulation of nearby glutamatergic afferents The top middle panel shows the location of the stimulation electrode in the LDT. Stimulation is adjusted until inward current with similar amplitude is consistently evoked (electrophysiological traces to the left). Following a 100 Hz, theta-burst stimulation (TBS) protocol (shown in the middle panel, see section “Methods” for more details), combined with a simultaneous depolarization of the recorded cells, the amplitude of the inward current was enhanced in these cells (electrophysiological traces to the right), which was a persistent effect, indicative of LTP, likely due to insertion of AMPA receptors into the postsynaptic membrane (top images).

The LDT was originally designated Ch6, based on presence of cholinergic neurons, however, this phenotype represents the minority of cells in this nucleus with GABA and glutamate neurons outweighing the numbers of cholinergic cells, and the three types exhibit some regional specificity across the rostral, medial, and caudal LDT (Wang and Morales, 2009; Luquin et al., 2018). The majority of cholinergic cells can be identified by the combination of a polygonal-shaped large soma, and presence of an A-type K^+^ current without/with a T-type Ca^+2^ current, defined as type III or II neurons, respectively (Leonard and Llinás, 1990). Small, round-shaped neurons with exclusive presence of T-type Ca^+2^ current are putatively GABAergic neurons, and are classified as type I. Glutamate cells are intermittent in size, albeit mostly smaller than cholinergic ones (Boucetta and Jones, 2009). We have recently confirmed the high-degree of correlation between soma size, and presence of A- or T-type currents, with phenotype in the LDT (Polli and Kohlmeier, 2019). In a protocol employing minimum external stimulation designed to reveal silent synapses, presence of synapses with NMDA receptors and absence of functional AMPA receptors were found in three cell types in the juvenile LDT (Polli and Kohlmeier, 2019; Figure 2). Further, pilot studies found that while not present in every cell examined, LDT neurons from juvenile mice can exhibit increases in synaptic strength at glutamatergic synapses upon theta-burst stimulation (TBS; Figure 3). Although we did not conduct a systematic study that would have allowed us to compare response proportions, this effect did appear in Type I, Type II, and Type III LDT cells. As this form of LTP was elicited by coupling the TBS protocol with depolarization of the postsynaptic neuron (see section “Methods”), this suggests the possibility of a NMDAR-dependent LTP mechanism in these cells, a hypothesis which is currently under investigation in our lab. When taken together, these published and preliminary cellular findings have led us to conclude that glutamate receptor-involved, cellular processes resulting in alterations in synaptic strength exist within all phenotypes examined within the LDT. Further experiments combining high-resolution imaging, molecular quantification of protein synthesis, and electrophysiological recordings in optogenetically-identified neuronal subtypes would provide confirmation of these data, and allow a full characterization of the processes involved in this phenomenon.

### Evidence for Plasticity Induced Within the LDT by Exposure to Drugs of Abuse in the Postnatal Period

Development of drug dependency includes a large, learning component, albeit a maladaptive one. Plasticity mechanisms seen in other forms of learning have been shown in brain regions exposed to drugs of abuse and are believed to be involved in progression of drug-dependent behavior. When drugs of abuse are utilized, the entire brain is exposed and the LDT is no exception. Therefore, as plasticity is involved in the neural process leading to drug dependency, it would be of interest to know whether different classes of drugs that are abused and which have been shown to induce plasticity in midbrain and cortical regions, induce plasticity in the LDT. However, up until a few years ago, little was known regarding whether cellular actions can be engendered in the LDT by first-time, acute exposures to drugs which are commonly abused. As a step to elucidate the role the LDT plays in drug dependent behaviors at a cellular level, we have recently conducted *ex vivo* electrophysiological studies in order to assess the responsiveness of LDT neurons to acute exposures to different drugs of abuse associated with induction of plasticity in midbrain and cortex.

All the drugs of abuse which we tested *ex vivo* for acute cellular actions on LDT cells were shown to induce effects. Local applications of a cannabinoid 1 receptor (CB1R) agonist to cholinergic LDT cells elicited calcium release from intracellular calcium stores, and decreased the frequency of inhibitory postsynaptic currents (IPSCs) of LDT neurons, suggesting the functional presence of CB1Rs within the LDT nucleus which plays a role in induction of depolarization-induced suppression of inhibition (Soni et al., 2014). Further, acute bath application of cocaine or amphetamine was shown to elicit intracellular calcium rises in LDT neurons. This effect was abolished by prior exhaustion of intracellular calcium stores, and reduced in the presence of DA receptor antagonists (Lambert et al., 2017). In addition, nicotine excitation of LDT neurons has been demonstrated which was sufficient to induce firing, and rises in calcium in cholinergic neurons (Ishibashi et al., 2009).

As acute exposure to CB1R agonists and stimulants led to excitation of LDT cells, and since these drugs have been shown to induce plasticity in other brain regions, we have speculated that they could induce plasticity in the LDT, as there is functional evidence to support the conclusion that changes in synaptic strength do occur in the LDT which would alter output to target regions upon exposure to stimulants. Lesion of the LDT led to reductions in amphetamine behavioral sensitization, and chemical stimulation of the LDT 2 days after systemic amphetamine administration led to increases in glutamate levels in the VTA which was suggestive of LTP-like plasticity within the LDT-VTA circuit (Nelson et al., 2007). While not examined with amphetamine, although AMPA/NMDA ratios have been shown to be altered in the mesolimbic midbrain structures by relatively short term exposures to cocaine, short and longer term exposures did not alter this ratio in the LDT as examined *ex vivo* (Kamii et al., 2015). However, a 2-week exposure to cocaine in rats did reveal heightened presynaptic release of glutamate onto cholinergic LDT neurons (Kurosawa et al., 2013). Further, the increase was shown to involve NMDA receptor activation, production of nitric oxide and likely involved glutamatergic synapses derived from the mPFC. A similar effect on inhibitory transmission was not noted. However, reductions in GABAergic inhibition were induced by noradrenaline-mediated actions on presynaptic alpha-2 adrenoreceptors, which was an action only seen in cocaine-treated animals, suggestive of drug-induced presynaptic plasticity in the LDT involving the inhibitory system (Taoka et al., 2016). Repeated administration of cocaine was also shown to lead to an increase in firing rate of cholinergic LDT neurons, that was attributed to enhancement of a persistent sodium channel, which is considered a form of intrinsic plasticity (Kamii et al., 2015). Inhibition of this sodium channel abolished cocaine-induced CPP, suggesting the heightened excitability of the cholinergic cells was critical for encoding of motivational drive (Kamii et al., 2015). When taken together, the data suggest that in drug-experienced animals, enhanced membrane excitability in combination with presynaptic facilitation of glutamatergic drive results, and output of cholinergic LDT cells is heightened which could include output from the LDT in the VTA and NAc. Although the AMPA/NMDA ratio did not alter with cocaine (Kamii et al., 2015), based on *in vivo* LDT data with amphetamine (Nelson et al., 2007), and the mechanisms underlying plasticity induced by a variety of drugs of abuse in other neuronal regions (Bellone and Luscher, 2006; Nelson et al., 2007; Yuan et al., 2013; Pascoli et al., 2015), it could be speculated that under different drug exposure protocols, changes could occur postsynaptically in glutamate receptors in the LDT which could contribute to changes in excitability. Further, the excitatory drive would be facilitated if NA input, which would presumably be high during the stress of drug-seeking behaviors in drug experienced rodents, led to decreases in GABAergic inhibition via disinhibitory mechanisms reliant on NA tone (Taoka et al., 2016). Unfortunately, to the best of our knowledge, cocaine is the only drug of abuse that has been shown to induce glutamate-receptor dependent plasticity, which has been examined for a similar effect in the LDT. However, as the glutamatergic synaptic machinery necessary for altering synaptic strength is present in the LDT, the effects of acute and chronic exposure to other drugs of abuse on glutamate-based changes in synaptic strength should be examined in the LDT. Nevertheless, we conclude that drugs of abuse lead to heightened activation of the VTA via indirect actions on the LDT, and further, we speculate that input to the reward circuitry from the LDT in response to drugs of abuse does not remain fixed, but the drug exposure shapes or leads to variations in input to the VTA or NAc, even when the stimuli remains the same, due to glutamate receptor-based, postsynaptic plasticity within synapses of the LDT.

### Evidence for Plasticity Induced Within the LDT by Drugs of Abuse in the Prenatal Period

Recent studies conducted in our laboratory have provided knowledge toward understanding of the flexibility that the LDT exhibits in neuronal trajectory dependent on the environment present during development. We have exposed pregnant mice to nicotine via their drinking water and by use of prenatal nicotine exposed (PNE) offspring, monitored the functionality of the LDT postnatally with implications for a role of the LDT in negative behavioral outcomes seen in those exposed to nicotine in the prenatal period, including a higher susceptibility for development of dependency to drugs of abuse (Polli and Kohlmeier, 2018, 2019). Our focus was on alterations in the juvenile brain, as this is the riskiest time for negative behavioral outcomes if drugs of abuse are utilized during this period in ontogeny, which is an association seen in both non-prenatally exposed individuals and those exposed prenatally to nicotine during gestation. Further, this period is when great changes are occurring in LDT neuronal maturation, with pruning of synapses and loss of total cell numbers in the LDT (Ninomiya et al., 2005). Accordingly, permutations of the normal progression of these processes would have a large impact on signaling saliency within reward-related areas later in life, especially if those changes rode on top of the ontologically-associated, high risk for dependency.

### Presynaptic Glutamate Release

Ionic currents resulting from activation of membrane-inserted, ionotropic receptors can be pharmacologically isolated, which allows determination of occurrence of specific neurotransmitter signaling, such as glutamate, in the form of spontaneous and miniature excitatory postsynaptic potentials (sEPSCs and mEPSCs), with mEPSCs amplitude and frequency reflecting effects of action potential-independent glutamate release. While the amplitude of such currents has been correlated with postsynaptic function of glutamate receptors, particularly AMPAR, examination of the frequency by which these currents occurs has been a common initial analysis approach to investigate alterations in the probability of glutamate release from presynaptic terminals. A higher frequency of postsynaptic currents predicts a greater degree of glutamatergic release directed to a neuron, whereas a reduced frequency of EPSCs likely reflects a reduced degree of active glutamatergic input to the recorded neuron.

In our work, we have found a reduced frequency of mEPSCs in putative cholinergic LDT neurons associated with PNE treatment, suggesting a lower probability of activity-independent glutamate release from presynaptic terminals within this nucleus (Polli and Kohlmeier, 2018). A more direct assay of presynaptic release employs a paired pulse stimulation protocol resulting in two evoked EPSCs (eEPSCs), elicited by an external electrode placed nearby a patch-clamped, recorded neuron. Under such conditions, glutamate release probability can be estimated by the ratio between the amplitude of the first and second eEPSCs, in which an increased ratio is observed under decreased glutamate release conditions. This phenomenon is called short-term facilitation, and can be mechanistically explained by the residual calcium hypothesis, in which calcium at presynaptic terminals resulting from the first stimulation builds up with that from the second, leading to a potentiation in the number of vesicles released from presynaptic terminals, and an enhanced postsynaptic response during the second stimulation, which reveals a reduced probability of glutamate release at the first stimulus (Malinow and Malenka, 2002). By having employed this protocol in PNE male juveniles, paired pulse facilitation was found in putative cholinergic LDT neurons, which was reflected as reduced glutamate neurotransmission within these cells (Polli and Kohlmeier, 2018). These findings are consistent with the interpretation that PNE treatment results in reductions in glutamatergic drive directed to postsynaptic cholinergic neurons within the LDT, which corroborates findings of a smaller frequency of mEPSCs seen in the PNE.

### AMPA Receptors

The ontological expression of ionotropic glutamate receptor subunits in the rodent brain has been well characterized for both AMPA and NMDA receptors, and identified changes in the balance of these subunits throughout the lifespan suggest they play different functional roles during development, in which their subunit switch represents a key step in the maturation of glutamate signaling (Roberts et al., 2009; Henson et al., 2012; Diering and Huganir, 2018). Among AMPARs, a central process in receptor maturation is the insertion of GluA2 subunits in the postsynaptic AMPAR during development, which contributes to the stabilization of synapses (Diering and Huganir, 2018). In contrast to what is understood about post-translational editing of mRNA coding for the other AMPAR subunits, GluA2 mRNA editing promotes a codon exchange in the pore-forming M2 segment, in which a glutamine codon in the GluA2 gene is substituted by an arginine codon (Sommer et al., 1991). This alteration has a dramatic impact on AMPAR function, as it impairs the calcium permeability of the receptor, reduces single-channel conductance, and decreases intracellular polyamine blockage (Washburn et al., 1997). Accordingly, AMPARs composed of GluA1/3/4 subunits, and lacking GluA2, display higher conductance, calcium permeability, and inward rectification of the receptor current-voltage relationship. These calcium permeable AMPARs (CP-AMPAR) are highly expressed during the first postnatal weeks within the cortex, hippocampus, and VTA (Kumar et al., 2002; Ho et al., 2007; Bellone and Mameli, 2011), but decrease in presence due to a relatively higher insertion of GluA2 subunits which occurs across age. Interestingly, a higher functional presence of the immature CP-AMPAR seems to be a common brain outcome following exposure to drugs of abuse, and has been reported after postnatal administration of cocaine, morphine, and ethanol (Bellone and Luscher, 2006; Billa et al., 2010; Purgianto et al., 2013; Hausknecht et al., 2015). We have recently reported that PNE is associated with electrophysiological alterations in AMPAR-mediated signaling in the LDT, that were suggestive of an enhanced functional presence of CP-AMPARs among putative cholinergic neurons within the LDT nucleus that was confirmed by a demonstrated greater action of the pharmacological blocker of CP-AMPAR, NASPM, on AMPAR-mediated currents in PNE LDT neurons (Polli and Kohlmeier, 2018). Thus, we concluded that PNE treatment was associated with enhanced functional presence of the immature CP-AMPAR within the large LDT neurons of juvenile mice, resulting in enhanced AMPAR-mediated signaling responsiveness, including a higher amplitude of mEPSCs.

### NMDA Receptors

NMDA receptors also show PNE-associated changes in the LDT. Interestingly, the subunits affected are the same ones implicated to play a large role in TBS-induced LTP, and in drug-induced LTP in other brain regions where this has been examined. The GluN2B NMDAR subunit has been suggested to play a central role in brain mechanisms of plasticity associated with several physiological brain functions, including learning and memory (Qiu et al., 2011). Studies have demonstrated that activation of GluN2B subunits is necessary for cortical plasticity (LTP) and fear contextual memory (Zhao et al., 2005). This subunit is also involved in synaptic plasticity within the amygdala and hippocampus (Berberich et al., 2007; Miwa et al., 2008). Further, studies using transgenic mice found that the overexpression of GluN2B subunits in the forebrain induces higher activation of NMDAR and facilitates expression of hippocampal LTP, which was associated with enhanced learning and memory in several behavioral tasks (Tang et al., 1999). Complementary results were seen in aged mice, in which memory was rescued by upregulation of GluN2B subunits (Brim et al., 2013). The key role of GluN2B subunits in cognitive processes is further supported by observations of downregulation of this subunit in neurodegenerative disorders that present with cognitive dysfunctions. Lower expression of GluN2B subunits was reported in a rat model of Parkinson’s disease (Dunah et al., 2000), and lower levels of this subunit were found in human postmortem brains from individuals diagnosed with Alzheimer’s disease (Sze et al., 2001; Mishizen-eberz et al., 2004). In our PNE model, we reported higher functional presence of GluN2B subunits within synaptic sites in small, Type I, putatively GABAergic LDT neurons in the PNE (Polli and Kohlmeier, 2019). Given the role of the GluN2B subunit in other regions, our data suggest that these cells might undergo a potentiated form of LTP, in addition to an observed enhancement of sustained depolarization upon synaptic NMDAR activation due to actions on current kinetics conferred by the presence of GluN2B. Further, we saw a reduced functional presence of this subunit in putatively cholinergic LDT neurons (Polli and Kohlmeier, 2019), which could predict impairments or attenuated LTP induction within this cell population in PNE juveniles.

The GluN3 subunit plays a vital role in the maturation of NMDARs and supports stabilization of glutamate signaling. Expression of this subunit increases and becomes widespread during the first postnatal days, but declines to very low levels throughout the lifespan (Pachernegg et al., 2012). In addition, this subunit is found at the postsynaptic density at PND8, but moves to perisynaptic and extrasynaptic sites shortly after and is not seen at postsynaptic domains during juvenile stages (Pérez-Otaño et al., 2006, 2016; Henson et al., 2012). GluN3A-containing NMDARs are characterized by a fast, desensitizing current and low calcium conductivity (Sasaki et al., 2002; Henson et al., 2010; Grand et al., 2018). We have reported that large, type II + III PNE LDT neurons, which likely include a majority of cholinergic neurons, displayed NMDAR kinetic activity typical of receptors containing GluN3A subunits, including reduced inward current and smaller intracellular calcium rises upon NMDA application, as well as a faster desensitization when agonist was applied in a manner designed to facilitate extrasynaptic receptor activation. While our data strongly suggested a higher functional presence of GluN3A subunits in these neurons, unfortunately, the lack of commercialized drugs that could specifically bind GluN3A sites (Pérez-Otaño et al., 2016) was a hindrance to definitively examining presence of this subunit. However, we believe that alterations in the GluN3A subunit in the PNE underlie our findings, as we were able to directly eliminate a role for the other potential subunit that could explain our data which is also found extrasynaptically, the GluN2D (Polli and Kohlmeier, 2019). As further support of our hypothesis of a heightened functional presence of GluN3A, expression of this subunit predicts GluA2-lacking AMPAR insertion at postsynaptic densities (Roberts et al., 2009; Yuan and Bellone, 2013), which is consistent with our findings of an enhanced GluA2-lacking functionality in the PNE. When taken together, we hypothesize that GluN3A subunit expression is altered in the PNE in putative cholinergic neurons. An enhanced insertion of GluN3A subunits following drug exposure would not be novel, since postnatal cocaine administration was shown to exert such alteration in DA VTA neurons (Yuan et al., 2013). Interestingly, prolonged expression of GluN3A subunits has been associated with higher liability for drug dependence, as well as cognitive and motor disabilities that have also been shown in humans to be associated with a higher risk following gestational nicotine exposure (Liu et al., 2009; Roberts et al., 2009; Spanagel, 2009; Marco et al., 2013; Yuan et al., 2013; Jin et al., 2014; Kehoe et al., 2014; Yang et al., 2015; Pérez-Otaño et al., 2016).

### Silent Synapses

Exposure to drugs of abuse has been shown to reactivate plasticity mechanisms seen during development in addiction-associated brain regions, which may play a role in the formation of addiction-related memories (Dong and Nestler, 2014) as reactivation of silent synapses remodels brain circuits and alters behavior, including those which are addiction-related (Brown et al., 2011; Lee et al., 2013). Higher rates of silent synapses in the rodent brain have been associated with cocaine, alcohol, nicotine, and morphine administration, which was an effect found mostly in NAc GABAergic populations (Huang et al., 2009; Graziane et al., 2016; Xia et al., 2017; Beroun et al., 2018). Our laboratory was the first to show functional evidence that LDT neurons of juvenile mice display silent synapses (Figure 2), and, further, that PNE treatment has an impact on their expression. While no differences were seen in silent synapses in cholinergic neurons following PNE treatment, an increased number of silent synapses were seen in putative GABAergic LDT neurons, which parallels the enhanced silent synapses seen in inhibitory NAc neurons following postnatal drug administration (Xia et al., 2017). The higher number of silent synapses seen in putative GABAergic LDT neurons associated with PNE treatment provides indirect support of the interpretation that PNE is associated with a delayed maturation of glutamate synapses in these inhibitory neurons. Further, since it has been previously shown that brief, local applications of nicotine in Schaffer collateral-CA1 connections convert silent synapses into active ones in newborn rat brain slices (Maggi et al., 2003), and previous studies from our group have demonstrated that nicotine has a strong excitatory effect on LDT neurons during the postnatal period (Ishibashi et al., 2009), it is tempting to hypothesize that postnatal nicotine, or exposure to other dependency-inducing drugs, could unsilence these synapses in the GABAergic LDT population following PNE treatment.

### Intrinsic Plasticity: PNE and the Action Potential

The intrinsic properties of neurons underlying neuronal excitability have been shown to exhibit plasticity in an activity-dependent fashion (Zhang and Linden, 2003; Lisman et al., 2018; Debanne et al., 2019). This process has been shown to occur in the LDT following postnatal chronic exposure to cocaine (Kaneda, 2019). Our studies provide further support that intrinsic plasticity can be altered by experience, as we showed that a form of intrinsic plasticity is engendered by early-life exposure to nicotine, as reflected in alterations in membrane properties and action potential kinetics of LDT neurons in the PNE (Polli and Kohlmeier, 2018). Further, PNE treatment was associated with lower activity-dependent intracellular calcium rises in putative cholinergic LDT cells of juvenile mice, assessed by single-photon calcium imaging of neurons activated by high current injection (Polli and Kohlmeier, 2018), which was an effect attributed to a reduced functionality of voltage-gated calcium channels (VGCCs). This finding is consistent with kinetic alterations seen in the action potential of the PNE LDT found in two independent studies from our laboratory (Christensen et al., 2015; Polli and Kohlmeier, 2018), as lower calcium rises mediated by VGCCs predict smaller activation of calcium-dependent potassium channels, with subsequent broadening of the action potential half-width (Bean, 2007), which were characteristics of the kinetics of the action potential observed in PNE LDT cells. Changes in VGCCs leading to reduced activity-dependent calcium predict a reduced excitability of these neurons, which parallels previous studies showing that PNE LDT neurons displayed a higher rheobase which is the amount of current necessary to induce an action potential (Christensen et al., 2015). Consistent with this, in unpublished observations, we noted that while PNE LDT neurons displayed higher inward currents upon AMPA application in the absence of TTX, a significantly smaller percentage of neurons fired action potentials following AMPAR activation in the PNE group relative to the number of cells in which AMPA induced action potential firing in control. A lower functionality of VGCC in these neurons upon depolarization reconciles findings from earlier work from our group, in which application of AMPA was associated with reduced intracellular calcium rises in PNE LDT neurons, despite enhanced postsynaptic AMPAR-induced current amplitudes (McNair and Kohlmeier, 2015; Polli and Kohlmeier, 2018). In the earlier study, the AMPA-induced depolarization in PNE LDT neurons was likely associated with a reduced VGCC functioning, resulting in an overall lower intracellular calcium rise. Although the precise calcium channel subtype that was altered in the PNE was not elucidated, in the CNS, VGCC function mediated by different VGCC subtypes is associated with neuronal development, membrane oscillation, and neurotransmitter release, among other physiological functions (Catterall, 2011; Simms and Zamponi, 2014). Given their role in all of these processes, early life dependent changes in their properties would be expected to profoundly affect communication within the LDT and transmission to target regions.

## Functional Significance of Alterations in LDT Development Associated With PNE

In LDT neurons, PNE was associated with alterations in AMPA and NMDA receptors, reductions in glutamate release and activity-dependent intracellular calcium, changes in intrinsic properties, and reductions in firing excitability, which would certainly impact on output to target regions, such as those within the mesoaccumbal pathway. One prominent hypothesis of drug dependence is the hypodopaminergic hypothesis which posits that those with relatively lower DA tone in the mesoaccumbal circuit exhibit a heightened sensitivity to drug stimuli, and are therefore more vulnerable to the reinforcing properties of drugs of abuse. PNE has been associated with a high liability for development of drugs of abuse, and the enhanced susceptibility for development of dependency to drugs of abuse associated with these individuals has been suggested to be due to hypodopaminergic VTA functioning resulting from the early life nicotine exposure (Navarro et al., 1988; Muneoka et al., 1997, 1999; Alkam et al., 2013; Yochum et al., 2014). In support of this supposition, DA VTA neurons exhibited significant alterations in burst firing in the adolescent PNE (Dragomir et al., 2017). Changes seen within the LDT following gestational nicotine exposure would be predicted to result in differential cellular responses to postnatal exposures to nicotine, and other dependency-inducing drugs. When taken together, our findings predict that upon LDT excitation, there would be a lower cholinergic tone within the VTA in PNE individuals when compared to individuals without these alterations. Further, if the uncovering of silent synapses in the GABAergic LDT population is induced by postnatal exposure to drugs of abuse, such as nicotine, is found to occur, and if drug-induced LTP occurs in these cells, the balance of inhibitory output to GABAergic target regions within the LDT and outside the LDT would be shifted, likely resulting in a net reduction in excitatory drive directed to DA-VTA cells, which would be exacerbated in the PNE as the number of silent synapses is greater. Given the modulatory role exerted by LDT on midbrain dopaminergic and striatal functions via control over burst firing, our findings which are suggestive of reduced cholinergic output in the PNE provide a neuronal basis for the alterations in burst firing of VTA cells seen in the PNE. These changes would be expected to lead to reduced release of DA in the NAc. Therefore, dysregulation in the LDT could play a significant role in the higher risks of negative behavioral outcomes in this population.

## Conclusion

The LDT had long been thought to be a hardwired “way station,” playing a role as a faithful effector, passively transmitting information to its targets, without any meaningful alteration of the informational content of signaling received from higher-order brain regions. Review articles focused on synaptic plasticity, including those detailing the neuronal plasticity engendered by drugs of abuse, remain centered on higher order structures involved in learning and memory with little to no mention of mechanisms underlying synaptic plasticity occurring in the brainstem, nor what the functional impact of such an alteration would be (Holtmaat and Svoboda, 2009; Lüscher and Malenka, 2011; Espinosa and Stryker, 2012; Kandel et al., 2014). Further, popular textbooks reflect that higher cognitive centers, including the limbic system developed on top of the brainstem in order to allow flexible behavior likely reliant on synapse rewiring (Millers, 2010; Dietrich, 2015). However, emerging information suggests that this interpretation does not reflect the potential for synaptic flexibility of the LDT, nor that this potential for a change in the strength of wiring could play a role in higher-order, cognitive behavior. The LDT is a dynamic nucleus, capable of functional alterations depending on environment-driven experiences, such as exposure to drugs of abuse. Behavioral and lesion studies suggest that the LDT does exhibit plasticity, with heightened excitatory output upon acute exposure to drugs of abuse. Chronic exposure to drugs of abuse can elicit pre and postsynaptic changes in LDT synaptic functioning, and mechanisms associated with long-term potentiation seem to be present at LDT synapses. Prenatal exposures to drugs of abuse alter the development of excitatory and inhibitory signaling in the LDT. Discovery of the machinery necessary for altering synaptic strength in the LDT leads us to conclude that it is possible that input to the reward circuitry from the brainstem does not remain fixed in the face of stimuli, but the experience of that stimuli could shape or vary input to the VTA or NAc, even when the stimuli remains the same, due to plastic changes in the brainstem.

As environmental experiences occurring in both prenatal and postnatal life have been shown to alter its functionality, it will be important to determine the impact of such alterations on drug dependence and other aroused, attentive, and motivated behaviors associated with prenatal nicotine exposure. Additionally, how these changes impact behaviors for which the LDT is more traditionally known to play a role, such as control of the sleep and wakefulness state and arousal, also warrant investigation. Further, additional studies should be conducted with different technical approaches such as optogenetics, electron microscopy, and characterization of early response genes and expression of exogenous molecules known to play a role in information processing-based synaptic alterations in order to confirm findings of plasticity in the LDT, as well as to determine which specific LDT neuronal subtypes can experience plastic changes. In summary, while arousal can be associated with compulsive or rote behavior, it can also be governed by flexible executive control. While such control is often believed to stem from cortical regions, we now know the brainstem could also play a role. Therefore, future studies should be conducted to provide a full characterization of the alterations in plasticity possible in the brainstem, and specifically within LDT neurons, as well as the underlying mechanisms and the behavioral consequences of plasticity-associated deficits promoted in this neural area by experiences throughout the lifespan if we are to truly understand cognitive-based processes including those leading to drug dependence and motivated and goal-directed arousal.

## Methods

### Animals

Animal procedures were authorized by the Animal Welfare Committee (Danish Ministry of Justice) and in accordance with European directives for the employment of rodents in research. All experimental procedures were conducted under valid permits (2014-15-0201-00031 and 2017-15-0201-01195). Naive NMRI male mice (Taconic, Denmark) between 11 and 15 days old were employed for *ex vivo* electrophysiological recordings. Animals were kept in the cage with their maternal progenitor under 12-hour light/dark cycle with lights on at 7:00, controlled humidity (52–62%), and constant temperature (22 ± 2°C). *Ad libitum* access to food and water was provided to the moms.

#### Brain Slices

Brain slices were obtained as previously described (Polli and Kohlmeier, 2018, 2019). Briefly, juvenile mice were anesthetized with isoflurane and decapitated. The skull was opened, and the brain was removed in ice-cold (0–4°C) artificial cerebrospinal fluid (ACSF) containing, in Mm: NaCl 124, KCl 5, Na_2_HPO_4_ 1.2, CaCl_2_ 2.7, MgSO_4_ (anhydrous) 1.2, Glucose 10, NaHCO_3_ 26; oxygenated in 95% oxygen/5% carbogen]. Coronal slices containing the LDT (300 μm) were obtained at 0–4°C with a Leica vibratome (VT1200, Leica, Germany), heated for 15 min at 37°C, and recovered at room temperature for 1 h prior to recordings. A sagittal cut was performed along the midline of the slice to separate the two hemispheres. Slices were placed in a microscope chamber (Olympus BX51WI, Germany, Olympus Europe) constantly perfused with oxygenated ACSF at 33°C with a controlled flow rate of 1 mL/min provided by 4 Head Perfusion pump (Ole Dick, Hvidovre, Denmark). Slices were visualized on screen via a CCD camera system (PCO Sensicam, Till Photonics, Germany) attached to the microscope using Live Acquisition software 2.2.0 (TILL Photonics, United States).

#### Whole-Cell Patch Clamping

Thin-walled borosilicate patch pipettes (ID: 1.1 mm, OD: 1.5; Sutter Instruments, United States; 7–12 MΩ) were fabricated on a horizontal puller (Sutter Instruments P-97) and filled with an internal solution (in mM: K-gluconate 144, MgCl_2_ 3, HEPES 10, NaGTP 0.3 and Na_2_ATP 4; EGTA free; 285 mOsm). The liquid junction potential was calculated as +14.7 mV at 20°C as previously described (Neher, 1992), and no correction was made. Voltage-clamp was performed with an Axoclamp 200B amplifier (Molecular Devices, United States) connected to an analog-digital converter (Digi-Data 1440A; Axon Instruments, Molecular Devices). Signals were sampled at 20 kHz and filtered with a 5-kHz low-pass Bessel filter with a scaled output gain of five. Pipette offset correction and fast capacitance compensation were employed before each cell recording. Series resistance and capacitance compensation (70–80%) were performed to decrease the transient response which could have introduced artifacts in the recordings. Only healthy neurons, with a stable holding current <200 pA were used. Access resistance was monitored during the experiments by the application of brief 5 mV pulses at each sweep, and only data obtained from patches with an access resistance maintained stably below 30 MΩ throughout the recording were considered. A combination of neuronal size and the presence/absence of the after-hyperpolarization A-type K^+^ or T-type Ca^+2^ currents were used to classify LDT neurons. A hyperpolarization voltage step (−85 mV) was followed by 7 depolarization voltage steps (−60 to −10 mV; Δ = +10 mV). To allow the employment of this protocol, QX-314 (5 μM; Alomone Labs, Israel) was added to the patch solution to block voltage-gated sodium channels. Small, round-shaped neurons with presence of T-type Ca^+2^ current, which are likely GABAergic neurons, were classified as type I. The medium/big-sized, polygonal-shaped neurons containing either A-type K^+^ current (type II) or both currents (type III), were likely cholinergic cells based both on previous literature (Leonard and Llinás, 1990; Kamondi et al., 1992; Boucetta and Jones, 2009). A recent study conducted in our group showed that cholinergic neurons were types II and III, whereas 90% of non-cholinergic neurons were type I (Polli and Kohlmeier, 2019). Although the combination of size and type is not a definitive method to classify LDT neurons based on their neurotransmitter release, we believe this simple approach is reliable in distinguish cholinergic from non-cholinergic neurons, with the caveat that glutamate cells could potentially be present within all neuronal types. D-Serine (10 μM; Sigma) was included as a co-agonist of NMDARs. All data were recorded using Clampex 10.3 (Axon Instruments) and analyzed using Clampfit 10.3 (Axon Instruments). Electrophysiological data were exported as text files and imported to Igor Pro 6.2 (Wavemetrics, United States) for figure preparation.

#### Induction of Long-Term Potentiation

In these pilot experiments, an external bipolar electrode (0.25 mm; FHC Microelectrodes, Bowdoin, ME, United States) used for presynaptic stimulation was placed in the ventral portion of the LDT nucleus (100–300 μm from the recorded neuron). The electrode was connected to a Stimulus Isolator A365 (World Precise Instruments, United States) with the output parameters controlled by a Pulse Stimulator Master-9 (A.M.P.I., Israel), triggered with TTL pulses generated by Clampex. 5 min after the acquisition of the whole-cell configuration (for equilibration), 10 pulses (0.033 Hz) were delivered to the slice via the stimulation electrode to record evoked excitatory postsynaptic currents (eEPSCs) with a voltage membrane command set at −60 mV, as a baseline. Intensity and duration of pulses were set to evoke EPSCs with amplitudes between 50 and 150 pA. Next, the patched neuron was depolarized to 0 mV, concomitant with the application of a theta burst stimulation (TBS) protocol, as previously described for the hippocampus (Kang et al., 1997). Four pulses (100 Hz) were injected 10 times (0.2 Hz). This protocol was repeated 4 times at 0.1 Hz frequency (see Figure 3 for detail). Following burst stimulation, the neuronal membrane was repolarized to −60 mV and kept under this voltage until the end of the recording. Between 20 and 30 stimulations (0.033 Hz) were employed to evoke EPSCs following the TBS protocol. A single neuron was recorded for each hemisphere of LDT-containing slices.

## Data Availability Statement

The datasets generated for this study are available on request to the corresponding author.

## Ethics Statement

The animal study was reviewed and approved by the Dyreforsøgstilsynet as mandated by the European Communities Council Directive (86/609/EEC).

## Author Contributions

Both authors did literature searching, data collection and/or interpretation, wrote and edited the manuscript.

## Conflict of Interest

The authors declare that the research was conducted in the absence of any commercial or financial relationships that could be construed as a potential conflict of interest.
